# Parental Nonstandard Work Schedules and Child Development: Evidence from Dual-Earner Families in Hong Kong

**DOI:** 10.3390/ijerph18105167

**Published:** 2021-05-13

**Authors:** Minseop Kim

**Affiliations:** Department of Social Work, The Chinese University of Hong Kong, Hong Kong, China; mskim@swk.cuhk.edu.hk; Tel.: +852-3943-7503

**Keywords:** parental employment, parental work, nonstandard work schedules, nonstandard hours, shift work, child development, child wellbeing, work-family conflict, Hong Kong, China

## Abstract

With the emergence of 24/7 economies, the practice of working nonstandard schedules has become increasingly common. This trend raises a concern about how parental nonstandard work schedules affect child development outcomes. Using data from dual-earner families with young children (age 5–6) in Hong Kong, this study examined the association between parental work schedules and child development. It also examined under what conditions parental nonstandard work schedules affect child development, with a focus on the moderating role of family income. Results showed that paternal nonstandard work schedules were negatively associated with overall child development. This association was particularly salient among low-income families. By contrast, maternal nonstandard work schedules were not associated with child development outcomes. These findings suggest that it is important to equip parents, in particular low-income fathers, to address challenges resulting from their nonstandard work schedules.

## 1. Introduction

Our way of life has undergone remarkable changes in recent decades. One of the chief reasons is a dramatic influx of mothers into the labor force [1], which has resulted in the rise of dual-earner families [2]. This change has prompted research interest in the interplay between parental employment and child development outcomes [2]. One aspect of parental employment that has been shown to influence child outcomes is time [3,4], which is a vital resource that parents invest in raising their children. Given that children fare better when parents have more time and can enjoy shared activities with them [5], considerable attention has been devoted to determining how parents allocate time between work and family responsibility [6]. The trend of increasing working hours for dual-earner families [7] has also led researchers to identify long working hours of parents as a potential risk factor for the positive development of their children [8].

Previous studies, however, have often assumed that nearly all parents work fixed, daytime schedules, and have tended to overlook the variety of temporal patterns in work hours, such as night/evening shift and irregular hours, which may have differential implications for child development. With the emergence of 24/7 economies, indeed, nonstandard work schedules (NWSs), defined as those outside the typical daytime span, have become increasingly common [9]. In the U.S., for example, about 20% of all wage and salary employees work in the evening, at night, on a rotating shift, or at irregular hours [10]. In Canada, approximately 30% of workers are employed on NWSs [11], while about 40 % of Australian employees regularly work NWSs [12]. NWSs are particularly common among low-income parents with young children [9]. This raises concerns about the possible influence of parental NWSs on young children’s developmental outcomes [13].

It is not clear *a priori* how parental NWSs affect child development. Work-family conflict theory, which highlights inevitable conflicts between the domains of work and family [14], suggests that parents working nonstandard hours are likely to experience time-based and strain-based conflict [15,16]. Given that NWSs may reduce the time overlap that parents share with their children at home, for example, parents may find it difficult to maintain regular parent-child interaction [17], which in turn may have adverse impacts on the children [18]. Evidence that NWSs may cause deleterious health effects such as sleep problems, chronic fatigue, and depression [19,20] also suggests that parents working nonstandard hours are likely to have less physical and mental energy for effective parenting [13]. By contrast, work-family enrichment theory posits that engagement in multiple roles across work and family may provide parents with resources that help them fulfil parental roles [21,22]. In particular, given that NWSs might be a preferable choice for some parents [23], such schedules are occasionally viewed as a family-friendly work options. For instance, evening or night work may allow parents, particularly those with preschoolers, to spend more time with their children during the daytime [24], which may promote positive child development outcomes [25].

The competing rationales suggest that it is important to conduct *empirical* research to determine the link between parental NWSs and child development. Therefore, a growing body of empirical investigation has emerged, but produced inconclusive results. For example, some studies have reported that parental NWSs were negatively associated with young children’s emotional/behavioral [15,26,27] and educational outcomes [28,29,30]. In contrast, other studies, including Ross-Phillips [31], Dunifon, et al. [32], and Morrissey, et al. [33], found no evidence that parental employment at nonstandard times were significant predictors of children’s various developmental outcomes. A further complexity is that, while the aforementioned studies tended to focus on maternal NWSs, recent studies examining both maternal and paternal NWSs among dual-earner families revealed the differential effects that maternal and paternal NWSs might have on child development [17]. For instance, Champion and colleagues [34] and Cho and Coulton [35] reported that paternal NWSs were adversely associated with children’s physical development [34] and academic outcomes [35], whereas such negative effects were not detected for maternal NWSs. Examining both maternal and paternal schedules not only sheds light on the under-researched effect of paternal NWSs, but also offers a methodological advantage, as studies [24,36] have found that mothers and fathers in dual earner families often make joint decisions about work schedules. In other words, maternal work schedules are correlated with paternal work schedules [17]. Therefore, failures to control for spouse’s schedules may produce biased estimates, underscoring the need to examine both maternal and paternal NWSs to paint the accurate picture of how parental NWSs affect children. 

The disparate findings may also suggest that the effects of parental NWSs on child development would vary depending on contextual factors, such as family income [13]. For low-income families facing economic hardship, NWSs are more likely to be an involuntary work option rather than a choice [23,37]. It is therefore likely that the adverse consequences of NWSs are more salient. By contrast, middle- or high-income parents who are more likely to voluntarily choose NWSs may take advantage of their positive side (e.g., maximizing parental time with children during daytime hours). They might also make use of their economic resources to buffer or offset the potential negative effects of NWSs [15]. In addition, the fact that low-wage jobs in which low-income parents are more likely to work provide less job autonomy and flexibility that may buffer work-family conflict resulting from NWSs [38], also suggests that NWSs might have more adverse effects on child development in low-income families. 

Previous studies in this area were primarily conducted in Western contexts, and thus little is known about whether and under what conditions parental NWSs affect child development in Asian contexts [35,39]. In Hong Kong, for example, the government has not produced official statistics on NWSs in the workforce, so even the prevalence of NWSs remains unknown. However, it is likely that a significant proportion of employees work nonstandard hours, as the recent growth in NWSs has been driven by the growth of the service sector [9], in which most Hong Kong people are employed. A survey also found that 35% of Hong Kong workers felt that they needed to be available 24/7 [40], suggesting that a substantial number of parents and children are likely to be influenced by NWSs. However, virtually no academic attention has yet been paid to the link between parental NWSs and children, despite its potential implications for Hong Kong families who have to juggle the competing demands of work and family [41]. To this end, this study sought to examine (1) whether maternal and paternal NWSs affect child development either negatively or positively, and (2) whether the effects of parental NWSs on child development differ by family income. The study used data from dual-earner families with young children (aged 5–6) in Hong Kong.

## 2. Methods

### 2.1. Data and Sample

A multi-stage sampling method was adopted to obtain a representative sample of dual-earner families with young children in Hong Kong. As a sample frame, the Hong Kong Government Education Bureau’s district preschool list was used, because it includes all registered preschools in Hong Kong. As around 90% of children aged 5-6 are attending preschools [42], a random sample drawn from the list is expected to approximate the population of preschoolers (age 5–6) and their parents in Hong Kong. As a first step, preschools were randomly selected from the list. Specifically, in an effort to ensure socioeconomic representativeness, Hong Kong’s 18 districts were first divided into three areas, based on the median household income of the districts (i.e., low-income districts [bottom six districts], middle-income districts [middle six districts], and high-income districts [top six districts]). Taking into account the number of preschools in the three areas, 13, 10, and 15 kindergartens were randomly selected from the low-, middle-, and high-income areas, respectively. Upon the approval of the preschools’ principals, the parents of the final-year students (5- to 6-year-olds) in the participating schools were invited to join the study. The survey questionnaire was then mailed to 615 families whose primary caregiver expressed interest in the study. Of those families, 521 returned the survey questionnaire in January or February 2019 (response rate = 84.7%). As the study’s aim was to examine both maternal and paternal work schedules, families that were identified as dual-earner families were selected for data analyses, which resulted in an analytic sample of 251 preschoolers and their parents in dual-earner families of Hong Kong. All of the participating families received a supermarket coupon (100 HK$ ≈ 12 US$) as a reward for their participation.

### 2.2. Measures

#### 2.2.1. Independent Variable (Parental Work Schedules)

Although many jobs today do not fall neatly into a few types of work schedule (e.g., working in the evening, at night, on a rotating shift), previous research has typically used self-reported classification approaches in which respondents are asked to choose a category that best reflects their *usual* work schedule [43]. This study also measured parental work schedules as a categorical variable, based on recommendations from Lambert and Henly [44], who reviewed various methods for measuring NWSs. Specifically, mothers and fathers were asked to indicate the schedule they usually worked at their primary job over the past 3 months, using the following six categories: (1) *standard* (if the job primarily begins at 8 a.m. or later and ends by 6 p.m.); (2) *evenings* (if an individual works primarily between 6 p.m. and midnight); (3) *nights* (if an individual works primarily between midnight and 8 a.m.); (4) *rotating shift* (if the work shift changes periodically from days to evenings or nights or vice versa); (5) *irregular or varying hours*; and (6) *others*. However, descriptive analyses revealed that such fine-grained categories resulted in small frequencies in some NWS categories. As in some of the previous studies [30,35], therefore, the categories of evening, night, rotating shifts, irregular hours, and others were grouped into one broad category of *nonstandard*, suggesting that a dichotomous variable of parental work schedules (standard vs. nonstandard) was primarily used for multivariate data analyses. As supplemental analyses, the effects of the fine-grained NWS categories were estimated in case the dichotomous variable of work schedules was found to be significant.

#### 2.2.2. Dependent Variable (Child Development Outcomes)

Child development outcomes were measured by the Chinese Readiness for School Scale (CRSS) [45], a holistic child development measure designed specifically for young children (age 4–6) in Hong Kong. This culturally-appropriate, 35-item scale assesses early child development outcomes in six domains, namely: 1) Language/Cognitive development (7 items; e.g., “Your child is able to retell stories”), 2) Social development (6 items; e.g., “Your child is able to help other children”), 3) Emotional/Behavioral development (5 items; e.g., “Your child does not push other children”), 4) General preparedness for school (8 items; e.g., “Your child is able to understand and follow the instruction”), 5) Self-management (3 items; e.g., “Your child is able to tidy up toys by him/herself”), and 6) Physical development (6 items; e.g., “Your child is able to fasten buttons properly”). The child’s primary caregiver was asked to rate their level of agreement with each item on a 5-point scale form 1 (strongly disagree) to 5 (strongly agree). The total score of the CRSS (i.e., the sum of the 35 items) was calculated and used as an indicator of children’s overall development, with higher scores indicating more positive development. Prior studies have demonstrated the reliability and validity of the scale in samples of Hong Kong preschoolers [45,46]. Cronbach’s alpha for the total score was 0.90 in this study.

#### 2.2.3. Control Variables

As previous research has shown the importance of family and child socio-demographic characteristics to both child developmental outcomes and parental work characteristics [23,47], an extensive set of family and child characteristics was controlled for. The child-related control variables included the child’s gender (male vs. female), age (5-year-old vs. 6-year-old), and ethnic background (Chinese vs. non-Chinese), and general health measured by parental response to a single item (“In general, would you say the child’s health is —?”) on a five-point Likert scale (5:excellent, 1:poor) [48]. The family characteristics included parents’ (mothers’ and fathers’, respectively) usual weekly working hours, highest education levels (<high school, high school, and >high school), and age (as of the survey year), family income (monthly), the total number of children under 18, the presence of a domestic helper at home (Yes vs. No), and the presence of one or more grandparents at home (Yes vs. No). 

### 2.3. Analysis

Linear regression was used to estimate the association between parental (maternal and paternal) work schedules and child development, controlling for an extensive set of family and child characteristics. To examine whether the associations differ by family income, the regression models were estimated respectively for subgroups defined by family income [*low-income families* (bottom one-third), *middle-income families* (middle one-third), and *high-income families* (top one-third] [39]. Interaction analyses were also conducted to test the statistical significance of the moderating effect of family income. Potential non-independence issues that may stem from the use of multi-stage sampling methods (i.e., children nested within preschools) were dealt with by Huber–White robust standard error [49]. In terms of missing data, each of the independent variables had just one or two cases with missing data, while four cases had missing data on the dependent variables. Given that the missing rates were not sizeable, complete data analyses (with listwise deletion) were conducted [50]. STATA 15 (StataCorp LLC, College Station, TX, USA) was used for data analyses. 

## 3. Results

### 3.1. Descriptive Statistics

Table 1 presents descriptive statistics of the sample. Of parents, 25.2% of fathers and 14.4% of mothers worked NWSs, while working irregular hours was the most common type of NWS for both mothers (9.6%) and fathers (9.6%). In terms of working hours, fathers worked longer than mothers (50.0 vs. 43.0 h per week. The average age was 41.4 years old (SD = 5.6) for fathers and 38.6 years old (SD = 4.4) for mothers, while approximately half of them had at least some college education (52.6% for mothers, 49.8% for fathers). Average monthly family income was approximately HK$66,000. About one-third of the families had one or more grandparents at home, while about half lived with domestic helpers. The children were predominantly identified as Chinese (96.8%). 52.6% were boys.

### 3.2. Regression Results

Table 2 displays results from the regression models in which child development outcomes measured by the CRSS scores were regressed on parental work schedules, controlling for family and child characteristics. Model 1, examining the broad measure of maternal and paternal work schedules, found that fathers’ NWSs were negatively associated with CRSS scores (b = ‒5.46, *p* < 0.05). By contrast, mothers’ NWSs had a non-significant association with CRSS scores (b = 3.12, *p* > 0.05). Given the significant effect that paternal NWSs had on child development, Model 2 examined the detailed measures of paternal work schedule as predictors. It found that children whose fathers primarily worked at night or in the evening had significantly lower CRSS scores than those whose fathers worked standard daytime hours (b = −15.43, *p* < 0.001). Similar negative associations were also detected for children whose fathers worked NWSs labelled as ‘Other’ (b = −11.93, *p* < 0.05). Among the control variables, family income was positively associated with child developmental outcomes (b = 0.21, *p* < 0.05). Gender, age, and health were also found to be significant predictors. Female children (b = 5.54, *p* < 0.05), 6-year-old (b = 5.89, *p* < 0.05) and healthy children (b = 7.44, *p* < 0.001) had better developmental outcomes.

**Figure 1:**
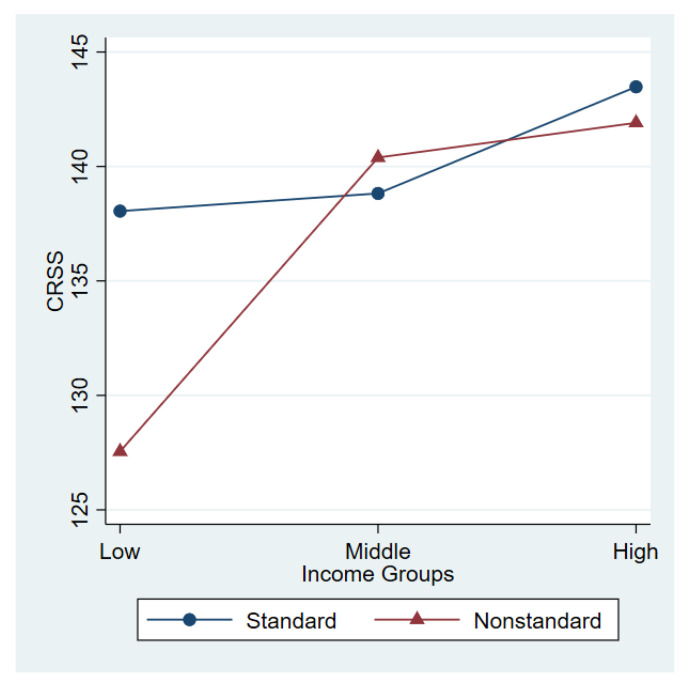
Predictive Margin of Father’s Work Schedule.

To examine whether the associations between parental NWSs and child development varied by family income, regression models were estimated separately for low-income families (the bottom third of family income), middle-income families (the middle third of family income), and high-income families (the top third of family income) (Table 3). As the sample size became smaller in the subgroups, we only examined the broad definition of parental work schedules (i.e., the dichotomous variable of parental work schedules), while retaining the control variables that were significant in the primary regression model (i.e., child gender, age, and health). The subgroup analyses found a negative association between paternal NWSs and child development (b = −10.42, *p* < 0.01) among low-income families, but no such association was observed in the case of middle- and high-income families. The interaction model in Table 4, including the interaction term between parental NWSs and family income categories, confirmed that the association between paternal NWSs and child development differed significantly between low-income families and middle-income families (the reference group) (Figure 1).

## 4. Discussion

As the 24/7 economy has evolved, working nonstandard hours has become common [9]. This significant shift in working patterns warrants empirical investigation of parental NWSs and their implications for young children who are at a crucial stage in their development [51]. The need for a better understanding of these implication is particularly pressing in Hong Kong, where work-family conflict is pervasive [41,52], but NWSs have rarely been viewed as a potential source of work-family conflict [53]. Using data from dual-earner families with preschoolers (age 5–6) in Hong Kong, this study sought to determine whether different maternal and paternal work schedules are associated with early child development outcomes. It also examined the conditions under which parental NWSs are associated with child development, with a focus on the moderating role of family income.

Descriptive analyses found that a substantial number of Hong Kong parents with preschoolers were employed on nonstandard hours. Specifically, 14.4% of working mothers and 25.2% of working fathers among dual-earner families were found to work nonstandard hours. These proportions are similar to those found in the United States [10], though they are not directly comparable due to the use of disparate samples and definitions of NWSs. The prevalence of NWSs in HK is also greater than those of other Asian countries (e.g., South Korea), as revealed by Cho and Coulton [35], who reported that 11 % of fathers and 10 % of mothers among dual-earner families of Korea worked NWSs. In terms of the types of NWSs, working irregular hours was prevalent for both mothers and fathers. Again, a similar pattern has been found in Western countries such as the United States [54]. These findings, though descriptive, are an important addition to our knowledge, suggesting that parents in Hong Kong, like their Western and Asian counterparts, encounter substantial demand for employment outside the traditional daytime hours, in response to the growth of the 24/7 economy. 

The multivariate models of the study provide empirical evidence that paternal NWSs may have adverse effects on early child development outcomes among dual-earner families. This offers support for the work-family conflict theory, which conceptualizes parental NWSs as a work characteristic that may have negative repercussions for family life [15,53]. Specifically, the CRSS scores of children whose fathers worked nonstandard hours were 5.46 points (or equivalently 0.29 standard deviation) below those whose fathers worked standard daytime hours. It therefore constitutes either a small or a medium effect, depending on the definition used in previous studies [55]. In results not shown but available on request, the standardized coefficient for family income was estimated to be 0.10, while the standardized coefficient for paternal NWSs was −0.13, which offers further proof that the effect of paternal NWSs is sizeable. However, not all parental NWS types were found to be significant predictors. The model analyzing the detailed definition of NWSs indicated that working evenings or nights had a significant negative effect. Though the small cell issue requires careful interpretation, this result is consistent with some prior research [15,56], which indicated that evening or night work is particularly detrimental to health and disruptive for the organization of family routines, such as family meals and bedtime routines, which leads to adverse consequences for child wellbeing. In addition to night/evening work, a work schedule labelled “Other” was also negatively associated with child development. Though it is not entirely clear what types of schedules led parents to choose this category, it suggests that some work schedules in Hong Kong’s changing world of work do not fall neatly into the NWS categories on which studies, including this one, have often relied, but such erratic schedules also pose challenges for parents and families. Further research is needed to understand the more exotic types of NWS and their implications for families in Hong Kong. 

It should be noted that the adverse effect of paternal NWSs was not evenly distributed across income groups. Moderation analyses, inclusive of subgroup and interaction analyses, found that the negative association between paternal NWSs and child development was salient among low-income families, but was not observed for middle and high-income families. Though Hong Kong is a developed economy characterized by material prosperity, income inequality and poverty have remained lingering problems [57]. Recent data have identified approximately 1.4 million people or a fifth (20.4%) of the total population as experiencing income poverty [58]. The city’s high cost of living, resulting primarily from the exceptionally high cost of accommodation, coupled with the relative lack of income-support programs [59], force many low-income workers to struggle to finance their family expenditure [57]. Under these circumstances, the nonstandard work arrangement for low-income parents in Hong Kong is likely to reflect the job demand or requirement of the low-wage labor market rather than an intentional or voluntary choice for balancing work and family responsibilities [23]. When working nonstandard hours, therefore, low-income parents are more likely to experience parenting stress [60] and unacceptable parenting behaviors [61], which in turn may adversely affect child development outcomes. Such challenges faced by low-income parents, coupled with their limited ability to afford children’s developmentally-enriching activities that may buffer the potentially negative effects of parental NWSs, may explain why the negative association of paternal NWSs with child development was more pronounced among low-income families.

It is equally important to highlight that, in contrast to paternal work schedules, maternal work schedules were found to be a non-significant predictor, suggesting a gender difference in the association between parental NWSs and child development. These results indeed align with those of some prior studies, which examined both maternal and paternal work schedules and reported similar patterns of gender difference when examining children’s educational [35] and health outcomes [34]. A possible explanation is that there would be a gendered pattern of how working parents allocate their time to paid work and child rearing. Studies have documented that despite the erosion of the male breadwinner and female caregiver model, mothers still play a primary role in child rearing [2,62]. Mothers do not sacrifice the time they spend with children, irrespective of their employment status. In an effort to maintain time involved with children, for example, working mothers often sleep fewer hours and reduce personal leisure time, to make up for the time spent at work [63,64]. This is also true of mothers working nonstandard hours, as a study by Bogen and Cherlin [65] found that mothers’ NWSs did not affect how much time they spent with children. In a comparative study of maternal and paternal time use, Enchautegui [66] even reported that mothers worked NWSs so that they could spend more time with their young children (aged 0–5) than mothers working standard hours, which may explain why this study found a positive, albeit non-significant, coefficient for maternal NWSs. By contrast, fathers working nonstandard hours were found to spend fewer hours with their preschool-age children than their counterparts working standard daytime hours [66]. Indeed, fathers often play a supplemental role in child care, especially in Hong Kong [67]. Therefore, fathers working NWSs may be under less pressure to compensate for their time at the workplace, suggesting father’s time for parenting and/or parent-child interaction may be more easily influenced by NWSs [68]. This may explain why paternal NWSs, unlike maternal NWSs, were associated with worse child development outcomes, though more research is warranted to explore other factors that may account for the gender difference (e.g., labor market characteristics and educational systems) [35].

### Limitations

This study has several limitations that should be noted. First, parents working NWSs and their children may represent distinct groups, indicating that selection bias is a concern in estimating the effects of parental NWSs on child development. To address potential selection bias, the multivariable models of the study controlled for an extensive set of family and child characteristics that may be associated with child development and parental work schedules. In addition, sensitivity analyses were conducted to examine whether the specification of the control variables (e.g., models with/without working hour control variables) and/or how the control variables are treated (e.g., treating child health as a continuous variable or categorical variable) alter the association between parental NWSs and child outcomes, revealing that the study findings are robust. Even with these extensive control variables and sensitivity analyses, however, it is still possible that families are different in other ways that this study cannot control for. Hence, caution must be used in making a causal interpretation of the study findings. Second, although previous studies found that specific types of NWSs can affect children differently [36], the dichotomous measure of NWSs was primarily used for data analyses, due to small cell issues that excluded the possibility of differentiating among the various types of NWSs, particularly when conducting moderation analyses. Though some studies suggest that the broad, dichotomous variable of work schedules is more accurate in terms of capturing the erratic and complex nature of NWSs in the contemporary labor market [27,30], further research is needed to understand how various types of NWS affect children. Third, it is also important to note that this study relied on cross-sectional data to examine how parents’ *current* work schedules affect contemporaneous measures of child development outcomes. However, child development is a dynamic process characterized by continuous change and growth. Therefore, child outcomes may also be influenced by prior experiences, including prior parental NWSs and/or the cumulative time parents worked NWSs [17,36]. Future research, particularly longitudinal data collection, is necessary to address this limitation. Fourth, this study used data from dual-earner families to analyze both maternal and paternal work schedules simultaneously and thereby obtain unbiased estimates for maternal and paternal NWSs. However, a drawback is that this study was unable to examine whether and how parental work schedules affect children in single-earner families, including single-parent families, who are likely to be more vulnerable to the negative effect of parental NWSs [13]. Therefore, the study findings should be generalized to dual-earner families with young children in Hong Kong. Future studies are indeed warranted to examine the effect of parental NWSs across family structures. Fifth, for subgroup analyses, this study divided families into three income groups (i.e., the bottom, middle, and top third of family income) to ensure that each subgroup contains roughly a third of families, and thereby avoid or minimize the small cell issues, when conducting subgroup analyses. However, there are other classification methods (e.g., quartiles or quintiles), which may lead to different conclusions. Therefore, sensitivity analyses were conducted to compare subgroup models based on different cut-off points. They revealed that the findings of the study’s subgroup analyses were robust, regardless of subgroup classification methods. Additional research is still warranted to further validate the findings of the study (e.g., subgroup analyses using cut-off points guided by Hong Kong’s poverty line estimates). Lastly, the study offered supermarket coupons (100 HK$), which might incentivize low-income families to participate in the study. Given that offering such rewards is a standard practice for survey-based research, it is less likely that the reward exerted severe undue influence on survey participation (e.g., the study sample disproportionally over-represents low-income dual-earner families). Nevertheless, the findings of the study should be interpreted with caution. 

## 5. Conclusions

Relying on data from dual-earner families with young children (aged 5–6) in Hong Kong, this study sought to investigate: (1) whether maternal and paternal NWSs affect child development, and (2) whether the effects of parental NWSs on child development differ by family income. Results revealed that paternal NWSs were negatively associated with overall child development. This association was particularly salient among low-income families. By contrast, maternal NWSs were not associated with child development outcomes. 

This study is the first to provide empirical evidence that parental NWSs may have a negative effect on child development in Hong Kong, and its findings have implications for policy and practice. While most developed countries have a norm of standard working hours, Hong Kong has no legal regulations on working hours [69]. Though policy discussions about the adoption of a statutory standard for working hours have emerged, it revolves largely around “the number of hours” rather than “timing of hours”; therefore, it is not surprising that Hong Kong has no specific policy regulating work schedules comparable to the European Union’s 1993 Working Time Directive, which asserts workers’ entitlement to a minimum daily rest period (11 consecutive hours per 24-h period) [70]. Furthermore, the expansion of nonstandard hour jobs has been driven in part by employers’ demand for efficiency and consumers’ demands for convenience [9,71], and thus it is questionable whether in the near future such policies that directly regulate the labor market can be implemented in Hong Kong, which is often characterized as a *laissez-faire* policy regime [72]. 

Under these circumstances, a more feasible approach may be to develop and implement intervention programs designed to preemptively mitigate the adverse consequences of parental NWSs. For instance, Workplace Triple P [73] could be a nascent parenting intervention that can effectively address challenges faced by parents working nonstandard hours. Triple P (Positive Parenting Program) is indeed a well-established parenting program, with a large evidence base for its effectiveness in preventing children’s development problems by promoting parenting-related knowledge, skills, and confidence among parents [74,75,76]. As a variant of Triple P that specifically targets employed parents, Workplace Triple P has been demonstrated to produce a range of positive outcomes, including enhanced parenting efficacy and reduced individual and work-related stress [73,77,78]. Although Workplace Triple P has not been used for parents in Hong Kong, an evidence base supporting the effectiveness of the original Triple P in Hong Kong [79,80] indicates that Workplace Triple P is also likely to be a promising option for working parents in Hong Kong, including those working nonstandard hours, while social service professional in Hong Kong are ready to implement the evidence-based parenting program [81,82]. Given the findings of the study, such parenting programs would be particularly beneficial to low-income fathers working nonstandard hours.

## Figures and Tables

**Table 1:** Descriptive Statistics.

Variables	Mean (SD)	%
Mother’s Work Schedule		
Standard		85.60%
Nonstandard		14.40%
(Night/Evening)		(2.80%)
(Rotation)		(2.00%)
(Irregular)		(9.60%)
Father’s Work Schedule		
Standard		74.80%
Nonstandard		25.20%
(Night/Evening)		(5.20%)
(Rotation)		(6.80%)
(Irregular)		(9.60%)
(Other)		(3.60%)
Mother’s Working Hours (per week)	42.97 (15.12)	
Father’s Working Hours (per week)	50.00 (15.91)	
Mother’s Age	38.56 (4.41)	
Father’s Age	41.35 (5.56)	
Mother’s Education		
<High School Diploma		11.16%
High School Diploma		36.25%
>High School Diploma		52.59%
Father’s Education		
<High School Diploma		18.47%
High School Diploma		31.73%
>High School Diploma		49.80%
Family Income (in 10,000 HK$)	6.59 (8.37)	
Number of Children	1.76 (0.61)	
Living with Grandparent(s)		31.20%
Living with Domestic Helper		51.20%
Child Gender (ref: Male)		52.59%
Female		47.41%
Child Age (ref: 5 years)		87.65%
6 years		12.35%
Child Ethnicity (ref: Chinese)		96.79%
Non-Chinese		3.21%
Child Health	3.32 (0.81)	
CRSS Score	138.93 (18.75)	
N	251	

**Table 2:** Regression Results.

Variables	Model 1		Model 2	
	b	RSE	b	RSE
Mother’s NWS (ref: Standard)	3.12	3.10	2.96	2.99
Father’s NWS (ref: Standard)	−5.46 *	2.61		
Night/Evening			−15.43 ***	4.18
Rotation			−3.05	3.96
Irregular			0.36	3.95
Other			−11.93 *	4.80
Mother’s Working Hours	−0.004	0.07	0.0003	0.07
Father’s Working Hours	−0.02	0.07	0.02	0.06
Mother’s Age	−0.23	0.29	−0.12	0.31
Mother’s Education (ref: < High School Diploma)				
High School Diploma	6.17	4.42	6.64	4.40
>High School Diploma	7.74	4.59	7.78	4.48
Father’s Age	0.27	0.27	0.20	0.27
Father’s Education (ref: < High School Diploma)				
High School Diploma	−3.08	3.33	−3.22	3.30
>High School Diploma	0.07	3.14	0.24	3.19
Family Income (in 10,000 HK$)	0.21 *	0.09	0.23 *	0.11
Number of Children	2.21	2.23	2.91	2.25
Living with Grandparent (ref: No)	−0.71	3.12	−1.40	3.18
Living with Domestic Helper (ref: No)	−0.61	2.69	−0.88	2.70
Child Gender (ref: Male)	5.54 *	2.47	6.44 *	2.54
Child Health	7.44 ***	1.54	7.38 ***	1.51
Child Age (ref: 5 years old)	5.89 *	2.84	6.06 *	2.88
Child Ethnicity (ref: Chinese)	6.66	6.69	6.76	7.01
Intercept	99.80 ***	15.65	94.97 ***	15.80
N	231		231	
R-squared	0.22		0.25	

* *p* < 0.05, *** *p* < 0.001; RSE: Robust Standard Error.

**Table 3:** Subgroup Analyses Result.

Variables	Model 1 (Low-Income)		Model 2 (Middle-Income)		Model 3 (High-Income)	
	b	RSE	b	RSE	b	RSE
Mother’s NWS (ref: Standard)	6.32	4.24	5.95	4.03	−3.78	5.06
Father’s NWS (ref: Standard)	−10.42 **	3.87	0.92	3.95	−1.72	5.25
Child Gender (ref: Male)	6.77	4.45	3.10	3.77	4.73	4.33
Child Health	6.71 *	2.59	8.55 **	3.03	7.62 **	2.53
Child Age (ref: 5 years old)	11.12 **	3.73	5.10	5.36	7.35	4.51
Intercept	109.91 ***	7.92	107.38 ***	10.59	115.44 ***	8.97
N	81		84		76	
R-squared	0.24		0.16		0.16	

* *p* < 0.05, ** *p* < 0.01, *** *p* < 0.001; RSE: Robust Standard Error.

**Table 4:** Interaction Analyses Result.

Variables	b	RSE
Interaction (Mother’s NWS x Family Income)		
Low	0.06	5.58
High	−10.44	6.48
Interaction (Father’s NWSs X Family Income)		
Low	−12.07	5.32
High	−3.14	6.12
Mother’s NWS (ref: Standard)	6.46	3.86
Father’s NWS (ref: Standard)	1.57	3.78
Family Income (ref: Middle)		
Low	−0.78	3.68
High	6.16	3.29
Child Gender (ref: Male)	4.60 *	2.29
Child Health	7.71 ***	1.48
Child Age (ref: 5 years old)	7.93 **	2.52
Intercept	108.14 ***	5.34
N	241	
R-squared	0.22	

* *p* < 0.05, ** *p* < 0.01, *** *p* < 0.001; RSE: Robust Standard Error.

## Data Availability

The datasets of the current study are not publicly available due to the fact that the datasets include information that could compromise the privacy of research participants. The data that support the findings of this study are available from the corresponding author, upon reasonable request.
